# Dental Treatment and Risk of COVID-19 in Japan

**DOI:** 10.3290/j.ohpd.b4342677

**Published:** 2023-08-29

**Authors:** Akifumi Enomoto, Yuto Takada, Yuko Kinoshita, Atsushi-Doksa Lee, Yasuhiro Kakiuchi, Takahiro Tabuchi

**Affiliations:** a Professor, Department of Oral and Maxillofacial Surgery, Faculty of Medicine, Kindai University, Osaka-Sayama, Japan. Conception, design, data acquisition, analysis, and interpretation, drafted and critically revised the manuscript. Gave final approval and agreed to be accountable for all aspects of the work.; b Associate Professor, Department of Oral and Maxillofacial Surgery, Faculty of Medicine, Kindai University, Osaka-Sayama, Japan. Conception, design, data acquisition, analysis, and interpretation, drafted and critically revised the manuscript. Gave final approval and agreed to be accountable for all aspects of the work.; c Assistant Professor, Department of Oral and Maxillofacial Surgery, Faculty of Medicine, Kindai University, Osaka-Sayama, Japan. Conception, data acquisition, analysis, and interpretation, drafted and critically revised the manuscript. Gave final approval and agreed to be accountable for all aspects of the work.; d Professor, Department of Forensic Medicine, Faculty of Medicine, Kindai University, Osaka-Sayama, Japan. Conception, design, data acquisition, analysis, and interpretation, drafted and critically revised the manuscript. Gave final approval and agreed to be accountable for all aspects of the work.; e Associate Director, Cancer Control Centre, Osaka International Cancer Institute, Osaka-Sayama, Japan. Conception, design, data acquisition, analysis, and interpretation, drafted and critically revised the manuscript. Gave final approval and agreed to be accountable for all aspects of the work.

**Keywords:** COVID-19, health concerns, infection control, oral medicine, special needs

## Abstract

**Purpose:**

During the Coronavirus Disease 2019 (COVID-19) pandemic, there has been concern about nosocomial infections acquired through dental practice, where machines – such as air turbines – that generate aerosols are used, and where there are many opportunities to come into contact with saliva and blood. Because there is no report to date on whether dental treatment is associated with a risk of SARS-CoV-2 infection in Japan, the aim of the present cross-sectional study was to examine the risk of SARS-CoV-2 infection associated with dental treatment.

**Materials and Methods:**

Cross-sectional data were gathered from the Japan COVID-19 and Society Internet Survey (JACSIS), a large-scale internet survey conducted in 2021 (n=28,175). From September 27, 2021, to October 30, 2021, the questionnaires were distributed to candidates selected from the panelists of a Japanese Internet research company to represent the Japanese population regarding age, sex, and residential prefecture using a simple random sampling procedure. The risk of SARS-CoV-2 infection related to dental treatment was examined and analysed.

**Results:**

Multivariable logistic regression analysis showed that younger age, male sex and living alone were statistically significant factors positively associated with SARS-CoV-2 infection, whereas the presence or absence of dental treatment was not statistically significantly correlated with SARS-CoV-2 infection.

**Conclusion:**

The present epidemiological study showed that dental treatment is not a positive risk factor for SARS-CoV-2 infection in Japan.

At the end of 2019, an outbreak of the new Coronavirus Disease 2019 (COVID-19) began in Wuhan, China, and spread around the world, causing an unprecedented public healthcare crisis.

After the first confirmation of COVID-19 in Japan in January 2020, the outbreak continued to spread nationwide through imported cases and domestic transmission. Coronavirus SARS-CoV-2 is a strain of the severe acute respiratory syndrome-related coronavirus (SARr-CoV). This emerging respiratory tract infection has resulted in a cumulative total of more than 33 million confirmed cases and more than 70,000 deaths in Japan as of 13 February 2023 (https://corona.go.jp/en/, COVID-19 Information and Resources, Cabinet Secretariat, Japan). The main transmission routes of the new coronavirus (SARS-CoV-2) are via droplets and contact.^22^ Additionally, the risk of infection by SARS-CoV-2 in aerosols has also been reported.^1,19,21,23^ Thus, the infectious COVID-19 virus contained in bioaerosols generated during dental procedures could be the main mode of transmission of SARS-CoV-2 during dental treatment. This working environment might place all dental care providers and their patients at an increased risk of exposure.

From the initial period during which SARS-CoV-2 infection became widespread, there has been concern regarding nosocomial infection through dental practice.^19^ The Japanese government declared the first state of emergency on 7 April 2020. In the same month, the Ministry of Health, Labour and Welfare implemented thorough, standard preventive measures and directed that all non-urgent dental treatment be postponed (Government office business contact on April 6). However, this communication did not indicate the criteria for postponing dental care or consider the concept of providing dental care based on the situation.^10^ Accordingly, patients were confused about proper dental care, and dental consultations decreased nationwide.^7^ However, it has been reported that for both patients and dental staff, the transmission rate of SARS-CoV-2 is low for dental care performed in a dental health setting.^3,18^ To date, no study has investigated whether dental treatment is a risk factor for SARS-CoV-2 infection in Japan. Therefore, the aim of the present study was to examine the risk of SARS-CoV-2 infection associated with dental treatment, using data from a large internet survey conducted in Japan (n = 28,175).

## Materials and Methods

### Study Participants and Setting

The present cross-sectional study used data obtained in the Japan ‘COVID-19 and Society Internet Survey’ (JACSIS) study. Questionnaires were distributed between 27 September 2021 and 29 October 2021, to 33,081 candidates selected from the panelists of a Japanese Internet research company (Rakuten Insight, Inc) as representative of the Japanese population with respect to age, sex, and residential prefecture, using a simple random sampling procedure. Responses were obtained from 22,838 people (response rate: 22,838/33,081 = 69%). A follow-up survey using the same questionnaire was conducted with additional panel members, yielding 8162 respondents, to obtain a total of 31,000 respondents. Respondent surveys were collected according to the population distribution of prefectures in Japan. All participants provided web-based informed consent at registration. After excluding respondents who provided invalid responses to the questionnaire, the data of 28,175 respondents (median age: 50 years, range: 16–81 years) were included in the analysis (Table 1).

**Table 1:** Summary of the characteristics of patients who completed the survey (n=28,175)

	Age in years	Number	Proportion (%)
	16–19	573	2.0%
	20–29	3626	12.9%
	30–39	4149	14.7%
	40–49	5454	19.4%
	50–59	4785	17.0%
	60–69	4882	17.3%
	70–79	4882	16.0%
	≥80	179	0.6%
**Age mean ± SD / median / range**	50.1 ± 17.0 / 50 / 16–81		
**Sex**	Male	13,870	49.2%
	Female	14,305	50.8%
**Living alone**	Yes	5910	21.0%
	No (two or more persons)	22,265	79.0%
**Homeowner**	Yes	19,531	69.3%
	No	8644	30.7%
**Educational attainment**	Less than high school	392	1.4%
	High school	7592	26.9%
	Technical college	6248	22.1%
	University or more	13,777	48.9%
	Missing	166	0.6%
**Dental treatment (in the past year)**	Yes	12,637	44.9%
	No	15,538	55.1%
**Dental treatment (in the past year)**	Yes (have had)	12,637	44.9%
	No (reason: COVID-19)	7160	25.4%
	No (reason: other than COVID-19)	8378	29.7%

SD: standard deviation.

### Statistical Analysis

The main outcome measure in the study was the respondents’ SARS-CoV-2 infection status. Individuals who chose the answer, ‘I have been diagnosed with SARS-CoV-2 infection in the past year’ were assigned to the SARS-CoV-2 infection group, and this was used as the primary outcome variable. The survey also contained items regarding the following six explanatory variables: age, sex, living alone, homeowner, educational attainment, and the following item about dental treatment, ‘Have you had a dental treatment in the past year?’ The possible responses were ‘Yes’ or ‘No’, with ‘No’ further subdivided into ‘I haven’t had dental treatment because of the COVID-19 pandemic’ and ‘I haven’t had dental treatment for a reason other than the COVID-19 pandemic’ (termed ‘because of the COVID-19 pandemic’ and ‘for a reason other than the COVID-19 pandemic’ in the subsequent additional analysis). Potential confounding variables (living alone, homeowner, and educational attainment) were assessed in the questionnaire as socioeconomic factors.^15^ To compare the two groups, the Mann-Whitney U-test and the Χ^2^ test were used for continuous and categorical variables, respectively. Data were collected and analysed using a combination of software packages, including Microsoft Excel (Microsoft; Redmond, WA, USA), SigmaPlot (Systat Software; Palo Alto, CA, USA), and EZR (Saitama Medical Center & Jichi Medical University; Saitama, Japan).^12^ The results are reported as means ± standard deviation (SD) unless otherwise indicated.

### Ethics Approval

The study was approved by the Bioethics Review Committee of the Osaka International Cancer Institute, Japan (approval no. 20084). All procedures performed in this study were in accordance with the Ethical Guidelines for Medical and Health Research Involving Human Subjects of the Ministry of Health, Labour and Welfare, Government of Japan, and with the 1964 Helsinki Declaration and its later amendments.

## Results

The results of this study are reported in accordance with the STROBE Statement – a checklist of items that should be included in reports of cross-sectional studies (www.strobe-statement.org).

The number of subjects analysed was 28,175 (13,870 men and 14,305 women), of whom 260 (0.96%) were validated as the SARS-CoV-2 infection group. Table 2 lists subjects’ background factors according to group, and summarises the results of the initial assessment by univariate analysis of individual risk factors for a diagnosis of SARS-CoV-2 infection. Median age was statistically significantly lower in the SARS-CoV-2 infection group (39 years; range, 16–80 years) than in the SARS-CoV-2 non-infection group (50 years; range, 16–80 years; p < 0.0001). In addition, the proportion of men was statistically significantly higher (p = 0.0002) in the SARS-CoV-2 infection group (60.8%) than in the SARS-CoV-2 non-infection group (49.1%). Living alone and non-homeowner showed positive associations with the diagnosis of SARS-CoV-2 infection. There was no significant association with educational attainment. Of the total number of subjects, 12,637 (44.9%) had dental treatment in the past year and 15,538 (55.1%) did not. The frequency of diagnosis of SARS-CoV-2 infection in the past year was 110 (0.87%) in the former group and 150 (0.97%) in the latter group (no statistically significant difference, p = 0.42). Not having dental treatment due to fear of SARS-CoV-2 infection was also included as a factor. There could be a risk of reverse causality that may balance the risk of infection arising during dental visits. That is, some people may not have attended because they were afraid of catching COVID-19. Therefore, for our analysis, we divided ‘had dental treament’ vs ‘did not have dental treatment’ into three groups: 1) had dental treatment; 2) did not have dental treatment because of the COVID-19 pandemic; and 3) did not have dental treatment for a reason other than the COVID-19 pandemic. This analysis revealed no statistically significant difference between factors for having dental treatment or not among the groups (p = 0.56).

**Table 2:** Evaluation of variables related to SARS-CoV-2 infection and dental consultation

	Age in years	Infection (+) (n = 260)	Infection (-) (n = 27,915)	p-value
	16–19	14 (2.4%)	559 (97.6%)	
	20–29	64 (1.8%)	3562 (98.2%)	
	30–39	53 (1.3%)	4096 (98.7%)	
	40–49	55 (1.0%)	5399 (99.0%)	
	50–59	41 (0.9%)	4744 (99.1%)	
	60–69	24 (0.5%)	4858 (99.5%)	
	70–79	8 (0.2%)	4519 (99.8%)	
	80–	1 (0.6%)	178 (99.4%)	
**Age mean ± SD**		40.6 ± 15.0	50.2 ± 17.0	<0.0001*
**Sex**	Male	158 (60.8%)	13,712 (49.1%)	0.0002*
	Female	102 (39.2%)	14,203 (50.9%)	
				
**Living alone**	Yes	82 (1.4%)	5828 (98.6%)	<0.0001*
	No (two or more)	178 (0.80%)	22,087 (99.20%)	
				
**Homeowner**	Yes	149 (0.76%)	19,382 (99.24%)	<0.0001*
	No	111 (1.3%)	8533 (98.7%)	
				
**Educational attainment**	Less than high school	5 (1.3%)	387 (98.7%)	0.604
	High school	75 (0.99%)	7517 (99.01%)	
	Technical college	52 (0.83%)	6196 (99.17%)	
	University or more	128 (0.93%)	13,649 (99.07%)	
	Missing	0 (0%)	166 (100%)	
				
**Dental treatment**	Yes	110 (0.87%)	12,527 (99.13%)	0.42
	No	150 (0.97%)	15,388 (99.03%)	
**Dental treatment**	Yes (have had)	110 (0.87%)	12,527 (99.13%)	0.56
**No dental treatment (reason)**	No (COVID-19)	73 (1.00%)	7087 (99.00%)	
	No (other than COVID–19)	77 (0.92%)	8,301 (99.08%)	

SD: standard deviation; *statistically significant difference.

The identified variables were then entered into a multivariate logistic regression model (Table 3), which identified age, sex, and living alone as statistically significant factors related to a diagnosis of SARS-CoV-2 infection, whereas dental treatment was not a statistically significant factor related to a diagnosis of SARS-CoV-2 infection. The data were examined by adding the factors related to the reasons for the absence of dental treatment, which showed that dental treatment was not a statistically significant factor related to a diagnosis of SARS-CoV-2 infection.

**Table 3:** Multivariable logistic regression analysis of factors related to SARS-CoV-2 infection

		Odds ratio	95% CI	p-value
**Age**		0.97	0.96–0.97	<0.0001*
**Sex**	Male			
	Female	0.61	0.47–0.79	0.0002*
**Living alone**	Yes			
	No (two or more)	0.66	0.49–0.89	0.007*
**Homeowner**	Yes			
	No	0.98	0.73–1.32	0.90
**Educational attainment**	Less than high school			
	High school	0.80	0.32–1.99	0.63
	Technical college	0.67	0.27–1.70	0.40
	University or more	0.54	0.22–1.33	0.18
	Missing	–	–	–
**Dental treatment**	Yes			
	No	0.87	0.68–1.12	0.28
		Odds ratio	95% CI	p-value
**Age**		0.97	0.96–0.97	<0.0001*
**Sex**	Male			
	Female	0.61	0.47–0.79	0.0002*
**Living alone**	Yes			
	No (two or more)	0.66	0.49–0.89	0.007*
**Homeowner**	Yes			
	No	0.98	0.73–1.32	0.91
**Educational attainment**	Less than high school			
	High school	0.80	0.32–2.00	0.63
	Technical college	0.68	0.27–1.71	0.41
	University or more	0.54	0.22–1.33	0.18
	Missing	–	–	–
**Dental treatment**	Yes			
**No dental treatment (reason)**	No (COVID-19)	0.89	0.68–1.21	0.46
	No (other)	0.85	0.63–1.14	0.29

CI: confidence interval: *statistically significant difference.

## Discussion

This is the first epidemiological study conducted in Japan to indicate that dental treatment is not a positive risk factor for SARS-CoV-2 infection. The main outcome measure used in the study was a diagnosis of SARS-CoV-2 infection. Participants diagnosed with SARS-CoV-2 infection within the past year from the survey time were assigned to the SARS-CoV-2 infection group, and this was used as the primary outcome variable. The explanatory variables were age, sex, living alone, homeowner, educational attainment, and the presence or absence of dental treatment in the past year.

The present study examined the epidemiological relationship between dental treatment and the presence or absence of new SARS-CoV-2 infection. According to a report by the Japanese National Institute of Infectious Diseases, the causes of the spread of SARS-Cov-2 infection in general medical institutions in Japan are failure to adhere to basic hand hygiene, inadequate/inappropriate personal protective equipment or clothing, inadequate standard precautions for patients in whom SARS-CoV-2 is not suspected, and inadequate zoning. It has been reported that these factors have caused patient-patient infection as well as patient-medical staff infection, resulting in infection clusters.^13^ In dental care, treatment takes place as the patient maintains an open mouth, and dental turbines and/or ultrasonic scalers are used that generate aerosols during treatment. A previous report indicated the possibility that this situation could provide a transmission route for COVID-19 in dental clinics and hospitals.^19^ It has also been reported that dental-care staff are at a higher than average risk of exposure to SARS-CoV-2 because infectious microorganisms can spread via dental procedures that use dental turbines and/or ultrasonic instruments. Other possible routes of transmission are direct contact with infectious microorganisms in the saliva droplets of infected patients, and direct contact with contaminated dental instruments or dental supply system.^14^ Accordingly, it is very important to examine the risk of SARS-CoV-2 infection in dental treatment. In the present study, the proportion of individuals who had been diagnosed with SARS-CoV-2 infection in the past year in Japan was around 0.90%–1.00% in all three of the examined groups. As no statistically significant association was found between dental treatment and the presence or absence of SARS-CoV-2 infection in Japan, we can conclude that dental treatment is not a positive factor for SARS-CoV-2 infection. Several studies conducted in countries other than Japan have examined the rates of cumulative prevalence of COVID-19 in dental patients, dentists, and other dental staff members.^2,3,11,18^ In agreement with the results of those studies, the present data indicate that oral-health care was not a positive risk factor for SARS-CoV-2 infection in Japan, although the data differ in location or subjects, compared to the reports among countries.

There are several possible reasons why oral-health care was not identified in the present study as a positive risk factor for SARS-CoV-2 infection in Japan. Independent of the COVID-19 pandemic situation, for many years, dental clinics in Japan have implemented thorough infection prevention measures on a daily basis. Droplets containing saliva and blood that are generated during dental treatment are known risk factors for infection. Therefore, strong infection control measures are required for the use of equipment such as air turbines, which generate aerosols, and for the numerous situations that bring staff in contact with patients’ saliva and blood.^2,6,20^ Dental treatment performed using high-speed rotary instruments produces a large amount of splatter and particles that can be contaminated by microorganisms originating from the oral cavity. It has been suggested that such particulate mists contain blood-based elements. We have previously reported that blood-contaminated materials have the potential to be suspended in air as blood-contaminated aerosol.^9^ In addition, our previous study showed that strict compliance with barrier precautions, including routine use of an operating gown and visor mask, is recommended whenever oral surgery is carried out with high-speed rotary instruments.^8^ In Japan, dentists are fully equipped with personal protective equipment (PPE), and strict infection control measures are followed by all dentists. These measures include wearing goggles, a mask, and gloves while using single-use equipment, and the use of single-use sterilized tools for patients, among others.^8^ The same infection control measures are followed by dental hygienists.^10^ Therefore, prior to the COVID-19 pandemic, standard precautions were already being applied appropriately during general dental treatment and during oral surgery by dental staff in Japan. Therefore, it is likely that these measures would have prevented transmission of the virus to or from the patient. Indeed, dentists in the USA continued to show a high level of adherence to enhanced infection control procedures in response to the ongoing pandemic, resulting in low rates of the cumulative prevalence of COVID-19 among dentists.^2^ In addition, air-replacement and air-cleaning machines are always installed and used in dental clinics, as the air is easily contaminated. Therefore, even in the case of a patient infected with the virus, further spread is unlikely. Furthermore, most dental clinics in Japan require an individual appointment for each patient, and these can be staggered to minimise the number of people gathered in the waiting room, thus reducing the opportunity for person-to-person spread of SARS-CoV-2. There has been no report of the occurrence of COVID-19 clusters in dental clinics in Japan since the start of the domestic pandemic in 2019.

The present subjects diagnosed with SARS-CoV-2 infection were significantly younger than those who were not. In the 3rd and 4th waves of the COVID-19 pandemic in Japan, the number of newly infected people per day was 6000 to 8000. In the 5th wave, however, this number exceeded 25,000. The more infectious and more severe ‘Delta strain’ was the dominant strain in the 5th domestic wave, which began to spread nationwide in July 2021. Of those newly infected during the period from 14–20 July, 47.6% were in their 20s and 30s. With this explosive increase in the number of people with new SARS-CoV-2 infections, the cumulative number of infection-positive cases in the three months of the 5th wave reached approximately 900,000. The infection status of this 5th wave could have affected the results of the present research^4,10,16^ and led to the statistically significant difference in age. The proportion of women diagnosed with SARS-CoV-2 infection was significantly lower than that of men, which may be related to previously reported sex differences in COVID-19 attitudes and behaviours.^5^ In Japan, women are more likely than men to work in various ‘social sectors’ that require face-to-face contact, such as services, retail, tourism, and hospitality. The government’s limitation and suppression of activity in these sectors as infection control measures may have resulted in the lower number of infected women than men in Japan. Furthermore, it is possible that young people living alone, with low incomes and unstable employment conditions, could have continued their social activities, which may have affected the findings of SARS-CoV-2 infection.^17^

The present study has several limitations. First, given the cross-sectional study design, causal relationships between variables could not be determined, as it is difficult to elucidate a causal relationship between SARS-CoV-2 infection and the presence or absence of dental treatment. Second, although participants were recruited to represent the Japanese population in terms of age, sex, and residential prefecture, the respondents of a web-based survey might not fully represent the Japanese population using self-reported information. The survey was developed to cover broad topics rather than focus on dental health-related issues, which led to some difficulties. The observation period between 27 September 2021 and 29 October 2021 corresponded to the period of the 3rd, 4th, and 5th domestic COVID-19 pandemic waves in Japan. Although SARS-CoV-2 infection is properly tested by PCR at hospitals, clinics, and/or public health centers in Japan, accurate data regarding SARS-CoV-2 infection at the time of the survey could not be gathered from all participants. Thus, the association between dental treatment and COVID-19 infection might have been underestimated. In addition, the number of people who died was not counted, which could also have resulted in underestimation. Because dental treatment had initially been considered a risk factor for SARS-CoV-2 infection, the risk of SARS-CoV-2 infection associated with dental treatment was included in the large survey from which the present data were obtained. The design of the present study was confirmed to be adequate and acceptable based on the statistical power calculated for verification of type-2 error. Thus, dental treatment does not appear to be a positive risk factor for SARS-CoV-2 infection in Japan. The present epidemiological study provides meaningful insights regarding future dental infection control that are relevant to government and dental/medical institutions, and emphasises that all dentists in Japan should continue to use sound knowledge and infection control measures to advance the oral health of their patients.

## Conclusion

The present investigation of the association between SARS-CoV-2 infection and dental treatment in Japan identified age, sex, and living alone as statistically significant factors related to SARS-CoV-2 infection, whereas a history of dental treatment was not. These findings suggest that dental treatment is not a risk factor for SARS-CoV-2 infection in Japan.
